# Complement activation in astrocytomas: deposition of C4d and patient outcome

**DOI:** 10.1186/1471-2407-12-565

**Published:** 2012-12-01

**Authors:** Katri Mäkelä, Pauli Helén, Hannu Haapasalo, Timo Paavonen

**Affiliations:** 1 Department of Pathology, University of Tampere Medical School, Tampere, Finland; 2 Unit of Neurosurgery, Tampere University Hospital, Tampere, Finland; 3 Department of Pathology, Fimlab laboratories, Tampere University Hospital, Tampere, Finland; 4 University of Tampere, School of Medicine, Biokatu 6, Tampere 33520, Finland

**Keywords:** Astrocytoma, Glioblastoma, C4d, Complement, Inflammation, Survival, Immunohistochemistry

## Abstract

**Background:**

C4d is a cleavage product of complement component C4 and is considered to serve as a marker for the site of complement activation. In this study C4d staining of grade I-IV astrocytic tumors was studied to explore if there is an association between complement activation and the grade of tumor, or patient survival.

**Methods:**

Tissue micro-array samples of 102 astrocytomas were stained immunohistochemically. The material consisted of 9 pilocytic astrocytomas and 93 grade II-IV astrocytomas, of which 67 were primary resections and 26 recurrent tumors. The intensity of C4d staining as well as extent of C4d and CD34 staining were evaluated. The intensity of C4d staining was scored semiquantitatively. The extent of the staining was counted morphometrically with a point counting grid yielding a percent of C4d and CD34 positive area of the sample.

**Results:**

The intensity and extent of C4d staining increased in grade II-IV diffusely infiltrating astrocytoma tumors in line with the malignancy grade (p = 0.034 and p = 0.016, respectively, Kruskal-Wallis test). However, C4d positive tumor area percentages were higher in grade I pilocytic astrocytomas than in grade II-IV diffusely infiltrating astrocytomas (p = 0.041, Mann–Whitney test). There was a significant correlation between CD34 positive and C4d positive endothelial area fraction in diffusely infiltrating astrocytomas (p < 0.001, Pearson correlation). In these tumors, the increasing intensity of C4d staining was also associated with worsened patient outcome (p = 0.014, log-rank test).

**Conclusion:**

The worsening of patient outcome and malignant progression of tumor cells seem to be connected to microenvironmental changes evoked by chronically activated complement.

## Background

Astrocytomas are CNS tumors originating from astrocytic glial cells or their precursor cells. They can be divided into four grades according to WHO criteria by the appearance of cell atypia, mitosis, microvascular proliferation and necrosis [1]. Grade I astrocytomas are called pilocytic astrocytomas. They are considered benign as they have a clear borderline and are generally slow growing. The biological background of pilocytic astrocytomas differs from grade II-IV tumors. Grade II-IV astrocytomas have a diffuse growing manner and lack a clear borderline. They are therefore referred to as diffusely infiltrating astrocytomas. These tumors often renew and proceed into more malignant grades. Glioblastoma multiforme is a grade IV astrocytoma, and the most prevalent form of astrocytic tumors in adult patients. Microscopically, glioblastomas can be distinguished from grade II-III astrocytomas by the occurrence of necrosis and microvascular proliferation. Glioblastomas have a vivid microvascular proliferation rate and this results often in abnormal, even glomeruloid microvascular growth patterns. This can sometimes be seen in pilocytic astrocytomas, as well. Benign pilocytic astrocytomas may also reoccur, if a remnant has been left behind during operation. However, they never turn into grades II-IV.

Complement is an innate component of the immune system. It can be initiated by three different pathways, including the classical pathway, lectin pathway and the alternative pathway [2]. Degradation of the first component of activated complement system yields an active enzyme that continues the cascade by cleaving the next zymogen of the cascade into a functioning enzyme. The follow-through of complement leaves behind inactive fragments of complement components. C4d is created when the complement control protein factor I inactivates C4b by cleaving it into C4c and C4d [3]. C4d remains covalently bound to the activation point. There is no apparent biological function associated with C4d alone [4], but due to its covalent binding and relatively long half-life, it can be considered as an activation marker of the classical and lectin pathways of complement. Only the classical and lectin pathways are known to involve C4d, whilst in the alternative pathway there is no cleavage of the C4 component [5].

C4d has to date been most widely surveyed on allograft tissue rejections. In allograft rejection studies, C4d positivity has been mainly seen in the cytoplasm of endothelial cells. Studies concerning tumors have shown that neoplastic cells and their extracellular matrix can also stain positive for C4d. For example significant C4d immunostaining has been reported in tumor cells of papillary thyroid carcinomas [6] and around neoplastic follicular dendritic cells in follicular lymphomas [7]. In the central nervous system (CNS) C4d staining has been observed in both glial and endothelial cells, but yet to our knowledge no study has been made to widely examine the appearance of C4d in astrocytic tumor tissue.

In this study, we examined complement activation in grade I-IV astrocytomas utilizing C4d immunohistochemistry. The purpose was to compare the appearance of C4d staining between different grades of astrocytomas and between primary and secondary resections of these tumors. Also complement activation on microvessel endothelium of astrocytomas was examined. One aim was also to assess via C4d immunopositivity if the extent of complement activation correlates with patient survival. Yin et al. have shown that the complement system activates more easily in stressed endothelial cells than cells under normal physiological conditions [8]. We hypothesize that the pathological blood flow caused by the abnormal microvascular patterns in astrocytic tumors with vivid microvascular proliferation activate complement and cause them to stain more strongly by C4d than astrocytomas with less vivid microvascular proliferation.

## Methods

### Study material

The study material consisted of 102 astrocytomas, of which 9 were grade I pilocytic astrocytomas and 93 were grade II-IV diffusely infiltrating astrocytomas [grade II (n = 21), grade III (n = 16) and grade IV (n = 56)]. Of the 93 diffusely infiltrating astrocytomas (grades II-IV), 67 were primary tumors and 26 recurrent. First, the astrocytoma specimens were fixed in 4% phosphate-buffered formaldehyde and processed into paraffin blocks. On the basis of H&E-stained slides, one neuropathologist (H.H.) evaluated the tumors according to the WHO 2007 criteria [1]. One histologically representative tumor region was selected from each sample specimen. Thus collected samples were mounted into multitissue blocks, which were constructed with a custom made instrument (Beecher Instruments, Silver Spring, MD, USA). The sample diameter of the tissue cores was 1000 μm. Also samples representing normal, non-neoplastic brain were included into blocks to serve as controls.

The samples were obtained from surgically operated patients at the Tampere University Hospital, Tampere, Finland, during 1983 to 2001. The tumors were radically resected if possible and most patients with grade III-IV astrocytomas also received radiotherapy. Adjuvant radiochemotherapy was not used in the period of the study. Mean patient age was 59 years, the youngest patient being 12 years old and the oldest 85 years old. Overall survival was known for 62 patients. Patient survival was examined in a five year follow-up study. The follow-up time started after primary resection of the astrocytoma and ended after five years of follow-up or if the patient died. The study protocol was approved by the Ethical Committee of Tampere University Hospital and the National Authority for Medicolegal Affairs of Finland.

### Immunohistochemistry

Two slides were produced from each multitissue paraffin block, cut at 5 μm thickness. Fully automated immunostaining was performed by a Ventana BenchMark LT Automated IHC Stainer (The BenchMark Series automated slide preparation system by Ventana Medical Systems, Tucson, AZ, USA). Ventana EZ Prep solution (catalogue No 950–100, Ventana) was used for deparaffiniztion. For epitope retrieval CC1: Tris -EDTA buffer pH 8.0 (catalogue No 950–124,Ventana) was used at 95°C to 100°C for 30 minutes. The slides were rinsed between steps with Ventana Tris-based Reaction buffer (catalogue No. 950–300, Ventana). Slides were incubated at 37°C for 32 minutes with a C4d specific rabbit polyclonal anti human C4d antibody (Biomedica Medizinprodukte GmbH & Co KG, Wien, Austria) using a 1:10 dilution. The staining kit used was the Ventana Ultraview DAB Detection Kit. A sample of a rejected kidney allograft served as a positive control for C4d immunostaining.

To compare the C4d endothelial staining extent with the overall tumor vascularity, the second slide from each multitissue block was stained with CD34 antibody. Lyophilized mouse monoclonal antibody, specific to the human CD34 molecule ( Novocastra Laboratories Ltd, Newcastle Upon Tyne, UK) was used as an endothelial cell marker. The dilution used was 1: 500.

### Morphometry

The extent of the staining reaction was determined by area fractions of the positively stained tumor samples, using a point counting grid [9]. In this method, the sample is viewed with a microscope at magnification x400, and the point counting grid is placed over the tumor sample and the points covering the sample are counted. A value was given to each sample using the formula ((Σ C4d-P)/(Σ Pt)) x 100, where “Σ C4d-P” is the number of C4d positive points of a tumor sample and “Σ Pt” is the number of all points of the grid covering the entire tumor sample. C4d positive tumor area fractions and C4d positive endothelium area fractions were counted separately. Necrotic areas were omitted from the analysis.

The CD34 positive area fraction of the tumor sample was counted using the same morphometric method as for the C4d fractions. CD34 was assumed to stain specifically the endothelial cell cytoplasm and therefore the value acquired from the morphometric count was considered to represent the percentage of the total microvessel area of the tumor sample.

For further analyses, the tumor samples were scored for the intensity of C4d positivity with scores from zero to three (C4d INT). Zero was given if the sample was negative, one if slightly positive, two if clearly positive and three if strongly positive. Both endothelial positivity and tumor cell positivity were taken into account.

The tumor samples were also divided into three groups according to the extent of the staining reaction on the grounds of the C4d positive tumor area fraction (C4d EXT). 1 = no positivity, 2 = 2% or less positive tissue, 3 = more than 2% positive tissue. The tissue samples were also grouped according to the C4d positive endothelial area fraction (C4d END). 1 = no positively stained endothelium was seen, 2 = 0.5% or less of the tissue area was positively stained endothelium, 3 = more than 0.5% of the tissue area was positively stained endothelium.

To statistically compare the C4d endothelial positivity to overall vascularity of the tumor, a variable was created in which the percentage of the C4d positive endothelial area fraction was divided by the CD34 tissue area fraction percentage.

### Statistics

The statistical analyses were performed using SPSS for Windows (Chicago, IL, USA). The significance of associations were defined using Kruskal-Wallis test, Mann–Whitney test and chi-square test. The significance of correlations was determined utilizing Pearson correlation. Kaplan-Meier curves and log-rank test were used in the survival analyses.

## Results

**Figure 1:**
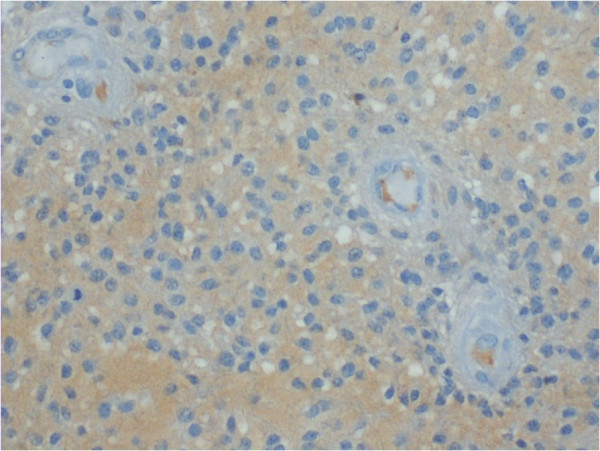
**Moderate diffuse cytoplasmic C4d immunopositivity in tumor cells of a grade II diffuse astrocytoma.** The endothelium of this tissue sample is negative. Magnification x200.

**Figure 2:**
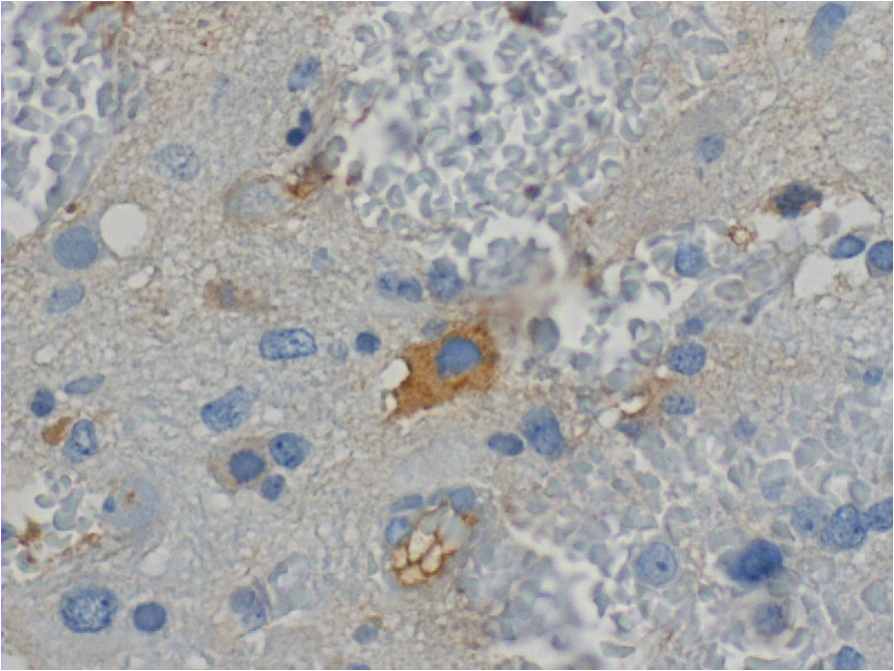
**Intensively C4d positive tumor cell surrounded by negatively stained cells in a grade III anaplastic astrocytoma.** Magnification x400.

**Figure 3:**
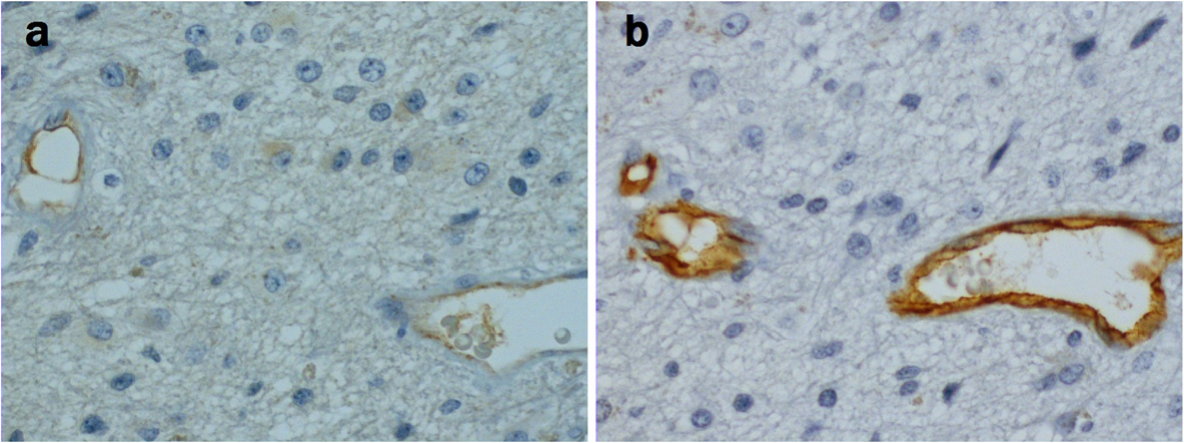
**a) C4d positive endothelium. b) CD34 positive endothelium.** The samples are from consecutive sections of the same grade III anaplastic astrocytoma. The sections are adjacent and the same capillaries are cut in both samples. Magnification x400.

**Figure 4:**
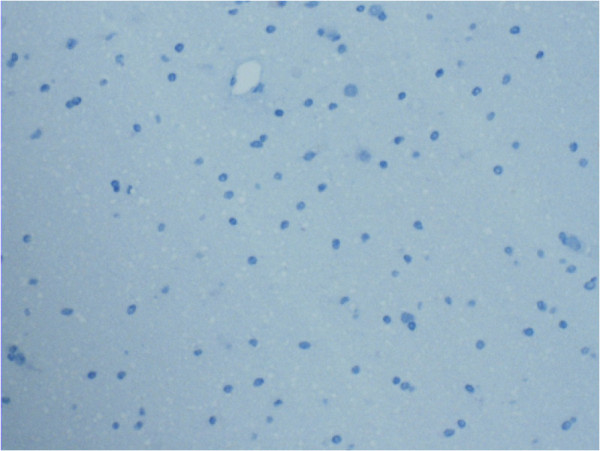
**Normal brain tissue which served as a control in C4d staining.** Brain tissue and endothelium in the capillary are C4d negative. Magnification x 200.

C4d positive staining was seen in endothelial cell cytoplasm, as well as in astrocytoma cell cytoplasm and tissue extracellular matrix. Positive staining is demonstrated in Figures 1, 2, 3. C4d negative normal brain tissue is demonstrated in Figure 4. Of the 102 astrocytomas (grades I-IV), 76 (74.5%) stained C4d positive. The C4d positive tumor area fractions of astrocytomas were between 0–84.7%, the mean value being 6.7%. C4d positive endothelium area fractions were between 0–3.5%, with a mean value of 0.2%. It was a common occurrence that tumor cells and endothelial cells simultaneously stained strongly.

In the grade II-IV diffusely infiltrating astrocytomas, the intensity of C4d staining (C4d INT) was found to correlate with the grade (p = 0.034, Kruskal-Wallis test, Table 1). The C4d positive tumor area fraction (C4d EXT) was also found to correlate with the grade of grade II- IV astrocytomas (p = 0.016, K-W test). Both intensity (C4d INT) and the C4d positive area fraction (C4d EXT) were found to increase progressively as the malignancy of the diffusely infiltrating astrocytoma advanced. The medians, means and standard deviations for C4d positive area fractions (C4d EXT) are presented according to the different grades in Table 2.

**Table 1:** Association between C4d staining intensity (C4d INT) and WHO grade*

		**Grades**				**Total**
		**Grade I**	**Grade II**	**Grade III**	**Grade IV**	
**C4d intensity**	**0**	1	10	4	11	26
	**1**	3	10	10	26	49
	**2**	3	1	2	16	22
	**3**	2	0	0	3	5
	Total	9	21	16	56	102

*(p = 0.034, Kruskal-Wallis test).

**Table 2:** The association of WHO grade to C4d positive tumor area, C4d positive endothelium, and to the fraction of C4d positive endothelium

**C4d EXT**	**Mean(%)**	**Std.(%)**	**Median(%)**	**p-value**
Grade I	18.4	22.5	8.2	0.041*
Grade II	2.1	8.7	0.0	
Grade III	0.7	0.9	0.4	
Grade IV	8.2	17.3	0.8	0.016**
**C4d END**				
Grade I	0.1	0.1	0.0	n.s.*
Grade II	0.1	0.2	0.0	
Grade III	0.3	0.5	0.0	
Grade IV	0.3	0.6	0.0	n.s.**
**C4d/CD34**				
Grade I	4.8	12.3	0.0	n.s.*
Grade II	3.0	7.5	0.0	
Grade III	12.1	19.1	0.0	
Grade IV	20.3	71.3	0.0	n.s.**

C4d EXT = C4d positive tumor area fraction, C4d END = C4d positive endothelial area fraction, C4d / CD34 = the proportion of C4d positive endothelial area fraction of the CD34 positive endothelium. p-value; * = Mann–Whitney U-test between pilocytic (grade I) and diffusely infiltrating (grades II-IV) astrocytomas; ** = Kruskal-Wallis test between different grades of diffusely infiltrating (grade II-IV) astrocytomas.

**Figure 5:**
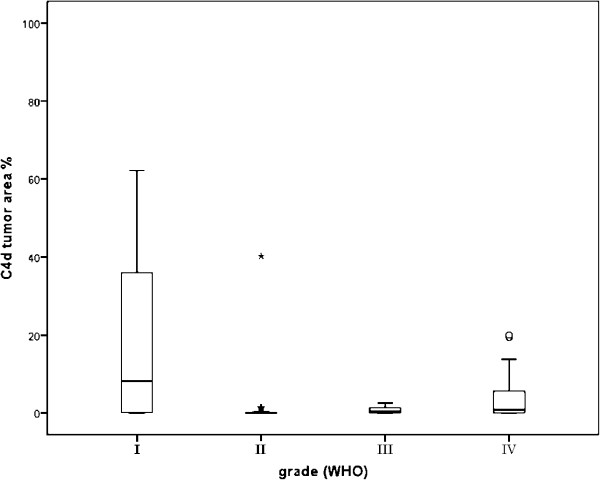
**Box-plot of C4d positive tumor area fractions (C4d EXT) in grade I-IV astrocytomas.** The C4d EXT groups: 1 = no positivity, 2 = 2% or less positive tissue, 3 = more than 2% positive tissue. (p = 0.041, Mann–Whitney U-test.).

There was a difference in the C4d staining between pilocytic grade I and diffusely infiltrating grade II-IV astrocytomas. The C4d positive tumor area percentages were higher in pilocytic tumors of grade I than in grade II-IV diffusely infiltrating astrocytomas (p = 0.041, Mann–Whitney test). This statistically significant difference is demonstrated in the box-plot in Figure 5. The mean of C4d positive tumor area fractions in pilocytic astrocytomas was 18.4%. The second highest positive tumor area percentages were achieved by the glioblastomas with a mean value of 8.2%.

A significant correlation between the CD34 positive endothelial area fraction and C4d positive endothelial area fraction was found in the diffusely infiltrating grade II-IV astrocytomas (r = 0.381, p < 0.001, Pearson Correlation). Figures 3a and 3b demonstrate the simultaneous C4d and CD34 positive staining of capillaries. As seen in Table 2, the proportion of C4d positive endothelium to overall vascularity (C4d / CD34) appears to rise along with the grade of the tumor, although no significance was achieved.

A difference in the C4d positivity was found between primary and recurrent diffusely infiltrating grade II-IV astrocytomas. Compared to primary tumors, recurrent tumors had more C4d positive samples in the C4d EXT variable. In 46% of primary tumors the C4d EXT was over 0%, whereas 65% of recurrent tumors had an C4d EXT value over 0%. (p = 0.047, Chi-Square test.). Comparing the other C4d variables between primary and recurrent tumors, no statistically significant differences were found.

### Survival

**Figure 6:**
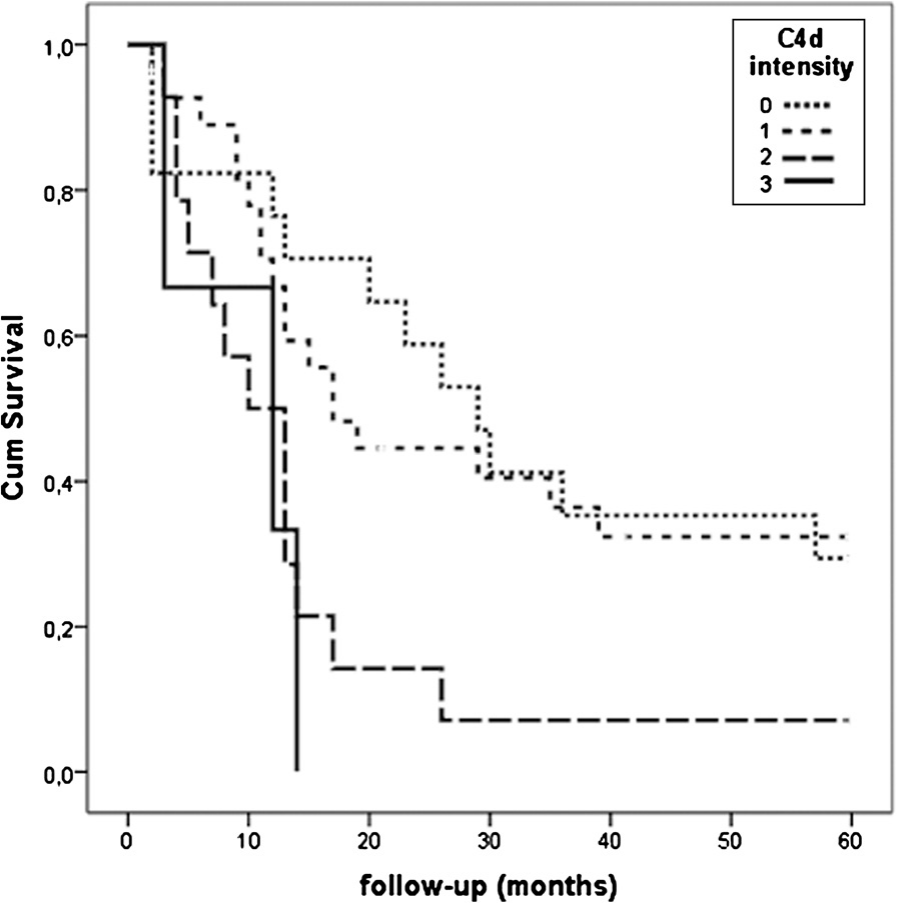
**Patient survival according to C4d staining intensity in diffusely infiltrating grade II-IV astrocytomas.** (p = 0.014, log-rank test).

In the five year follow-up, the increasing intensity of C4d staining (C4d INT) in diffusely infiltrating astrocytomas of grades II-IV was associated with patients having a worsened outcome (p = 0.014, log-rank test). The mean lifetime after primary resection for patients in the C4d INT group 0 was 32.5 months (95% confidence interval 21.9-43.0), in group 1 the mean lifetime was 29.8 months (CI 21.2-38.3), in group 2 the mean lifetime was 14.1 months (CI 6.7-21.4) and in group 3 the mean lifetime was 9.7 months (CI 3.0-16.3). The patient survival in different C4d intensity groups are presented in Figure 6. C4d EXT did not correlate significantly with survival. In addition, Cox multivariate analysis showed that only WHO grade was an independent prognosticator (Exp(B) 5.666, p < 0.001, 95% confidence interval 3.069 – 10.459). Other parameters included to the multivariate analysis were C4d INT, C4d EXT and patient age.

## Discussion

The intensity of C4d staining and C4d positive tumor area fraction were found to correlate with the grade of grade II-IV diffusely infiltrating astrocytomas. The increasing intensity of C4d staining in diffusely infiltrating astrocytomas was also associated with a patient’s worsened outcome in univariate survival analysis. Compared to primary tumors, recurrent tumors had more C4d positive cases. We also found a correlation between the CD34 positive area fraction and C4d positive endothelial area fraction in diffusely infiltrating astrocytoma samples. Interestingly, our results showed that in pilocytic grade I astrocytomas, the C4d positive tumor area fractions were higher than in diffusely infiltrating astrocytomas of grade II-IV. This was even though in the diffusely infiltrating astrocytomas positivity linearly and significantly followed the WHO grade. Although C4d intensity was found to be a significant prognosticator in univariate survival analysis in the present study, it did not appear as an independent prognostic factor in the Cox multivariate survival analysis. Reason for this is probably the close association of the C4d intensity with the tumor grade, which is found commonly to be the best prognostic factor of the diffusely infiltrating astrocytomas [1]. In addition, we believe that our material of primary diffuse astrocytomas is perhaps too small for reliable Cox analysis.

Vivid microvascular proliferation can be seen in pilocytic astrocytomas and glioblastomas [1]. One simple reason to the high C4d expression in these tumors could be that the abnormally proliferative, leaky blood vessels elicit inflammatory changes resulting in complement activation. Furthermore, the extensive activation of the complement in abnormal blood vessels may consume the serum C4 component and this way explain the study finding of Gousias et al., in which they showed a reduced serum C4 in patients with grade II-IV glioma and an inverse correlation between serum C4 and glioma grade [10]. However, in the case of pilocytic astrocytomas, the high C4d positive tumor area fractions could also be associated with complement inhibiting complement regulatory proteins (CRPs). There is evidence that benign tumors express CRPs in a much lesser extent than malignant tumors [11,12]. Thus complement activation could take place more readily in benign than malignant tissues due to a lower expression of complement inhibiting CRPs, leading to further emergence of C4d. Although, C4d could also accumulate on pilocytic cell surfaces, due to longer lifetime of the pilocytic cells compared to malignant astrocytic tumor cells.

In malignant astrocytomas CRPs have been connected to the ability to evade immune destruction. According to Mäenpää et al. malignant glioma cell lines are exceptionally resistant to complement mediated lysis, mainly because of the widespread expression of CRPs on their cell membranes [13]. Glioblastomas can also avoid complement-mediated killing by the active production of soluble complement inhibitors factor H and factor H-like protein 1 [14]. This may be also why glioblastomas stain more extensively than grade II and III diffusely infiltrating astrocytomas.

Hanahan and Weinberg have recently nominated the capability of cancer cells to evade immune destruction as a hallmark of cancer [15]. In the case of malignant astrocytic cells, CRPs could be one element that helps these cells evade immune destruction. Increased C4d intensity at the complement activation site may reflect thus the ineffectiveness of the immune destruction system against cancer cells, which leads to chronic activation of the complement system. Chronically activated complement can proceed towards inflammation [16]. Hanahan and Weinberg illustrate in their recent article that inflammation and genome instability are the enabling characteristics in the acquisition of core cancer hallmark capabilities, allowing cancer cells to survive, proliferate and disseminate [15]. Cytokines, free radicals, prostaglandins and growth factors are a part of the activated inflammatory complex. They can cause alterations in the creation of DNA mutations in tumor suppressor genes and failure in normal protein synthesis, causing defects in the function of the cell, leading to progression of cancer [17,18]. Understandably all this can lead to worsened patient outcome.

The univariate survival study results, which showed that a worsened outcome is associated with intensified complement activation in grade II-IV astrocytomas, can thus reflect the effect of the pathological microenvironment provided by inflammation. Also, since the inflammatory microenvironment allows cancer cells to survive, proliferate and make the cell genome unstable, it is possible that the inflammatory microenvironment could also enhance the recurrence of tumors after primary resection. In our study, recurrent diffusely infiltrating astrocytomas had more C4d positive cases than primarily resected ones, supporting this supposition.

## Conclusion

This study shows that the intensity of complement activation, detected by C4d positive staining, is associated with the grade of grade II-IV diffusely infiltrating astrocytomas and the primarity of resection. There is also an association between complement activation and the prognosis of patients suffering from diffusely infiltrating astrocytomas. The worsening of outcome can be connected to the microenvironment changes, which are caused by the inflammatory mediators evoked by complement activation.

## Abbreviations

CNS: Central nervous system; CRPs: Complement regulatory proteins.

## Competing interests

None of the authors declare a conflict of **c**ompeting interests.

## Authors’ contributions

All authors were involved in drafting the manuscript and approved the final manuscript. In addition KM carried out the immunohistochemical staining procedures and the morphometrical analysis of the study material, and participated in the statistical analysis. PH was involved in acquisition of the study material and revised the manuscript critically. HH was involved in acquisition of the study material, participated in the design of the study and performed the statistical analysis. TP participated in the design of the study and gave the final approval of the version to be published.

## Pre-publication history

The pre-publication history for this paper can be accessed here:

http://www.biomedcentral.com/1471-2407/12/565/prepub
